# Penetrating Wounds of the Abdomen

**Published:** 1904-05-07

**Authors:** 


### PENETRATING WOUNDS OF THE ABDOMEN.

In a paper read before the Chicago Surgical Society, and reported in the Annals of Surgery, Professor M. L. Harris brings forward some convincing reasons for early operation, in civil practice, in cases of perforating wound of the abdomen, as contrasted with the plan of waiting until symptoms pointing to serious visceral structures have ensued.

As he very justly points out, in the absence of hæmatemesis, hæmaturia, or passage of blood by the rectum, and provided that there is no escape of wounded viscera or their contents from the abdominal wound, there are no symptoms of perforation. And if it is decided to delay, it means that the surgeon is waiting for symptoms of hæmorrhage or peritonitis. It has been shown that in at least 95 per cent. of perforating wounds of the abdomen, damage which needs repair has been inflicted on some of the viscera. The 5 per cent. of unnecessary laparotomies performed, if Dr. Harris's principles are followed, will, in all probability be more than compensated for by the large number of lives that will be saved by timely interference. Thus he has had under his own care 16 cases of penetrating wounds of the abdomen with only three deaths. In one of these the fatal issue was due to profuse internal hæmorrhage, the patient dying before the source of hæmorrhage could be discovered and controlled. Another died from concomitant injuries to the spine and both lungs. These two cases may be fairly excluded, leaving fourteen with only one death—the patient in the fatal case succumbing to peritonitis, the result of eight visceral perforations. In two instances no visceral injury was found.

Although the numbers are small, the recoveries are over 90 per cent., as contrasted with the usual rate of 30 or 40 per cent., a degree of success which Dr. Harris attributes to the fact that all the cases but one were operated on within three hours of the time of the accident.

In concluding his paper, he emphasises the following points:—(1) In penetrating wounds of the abdomen there are no symptoms which indicate visceral injury, except those in connection with the stomach, urinary tract, and occasionally the lower bowel. (2) Apart from shock, all symptoms following such injuries are due to hæmorrhage or peritonitis. (3) To wait for symptoms of perforation of the intestine is to wait until peritonitis has developed. (4) Therefore every bullet or stab wound which penetrates the abdominal cavity should be operated on at the earliest possible moment. (5) No time should be wasted in attempting to demonstrate the presence or absence of intestinal perforation by such methods as rectal insufflation. (6) It is essential to systematically examine the entire gastro-intestinal canal in all cases, regardless of the point of entrance of the wounding body. (7) Whenever the alimentary canal has been perforated, suitable drainage should be employed.

